# Supplementary figures and images for: SF3B1 mutation–mediated sensitization to H3B-8800 splicing inhibitor in chronic lymphocytic leukemia
Source: Life Sci Alliance. 2023 Aug 10;6(11):e202301955. doi: 10.26508/lsa.202301955 (PMC10415613; doi:10.26508/lsa.202301955)

## Source Data For Figure 1

1C *SF3BI* WT

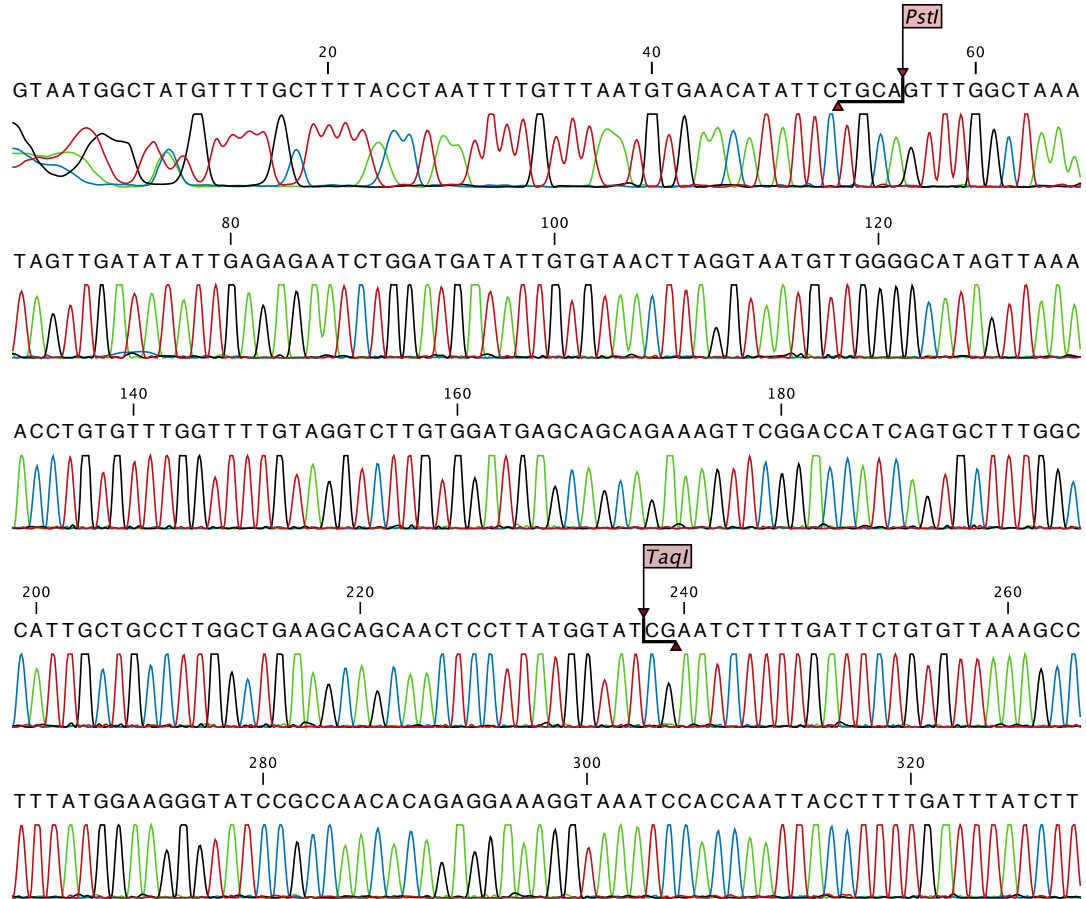

1C *SF3BI* K700K

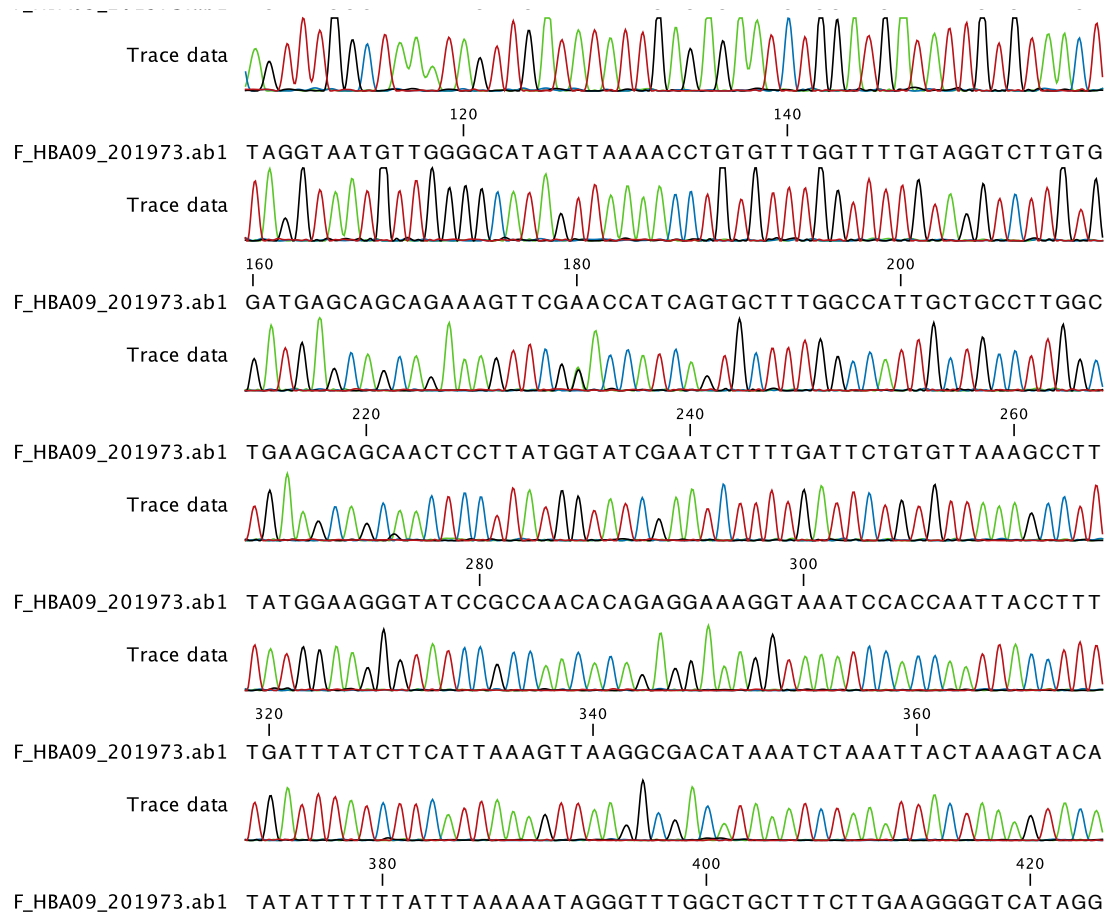

1C *SF3B1* K700E

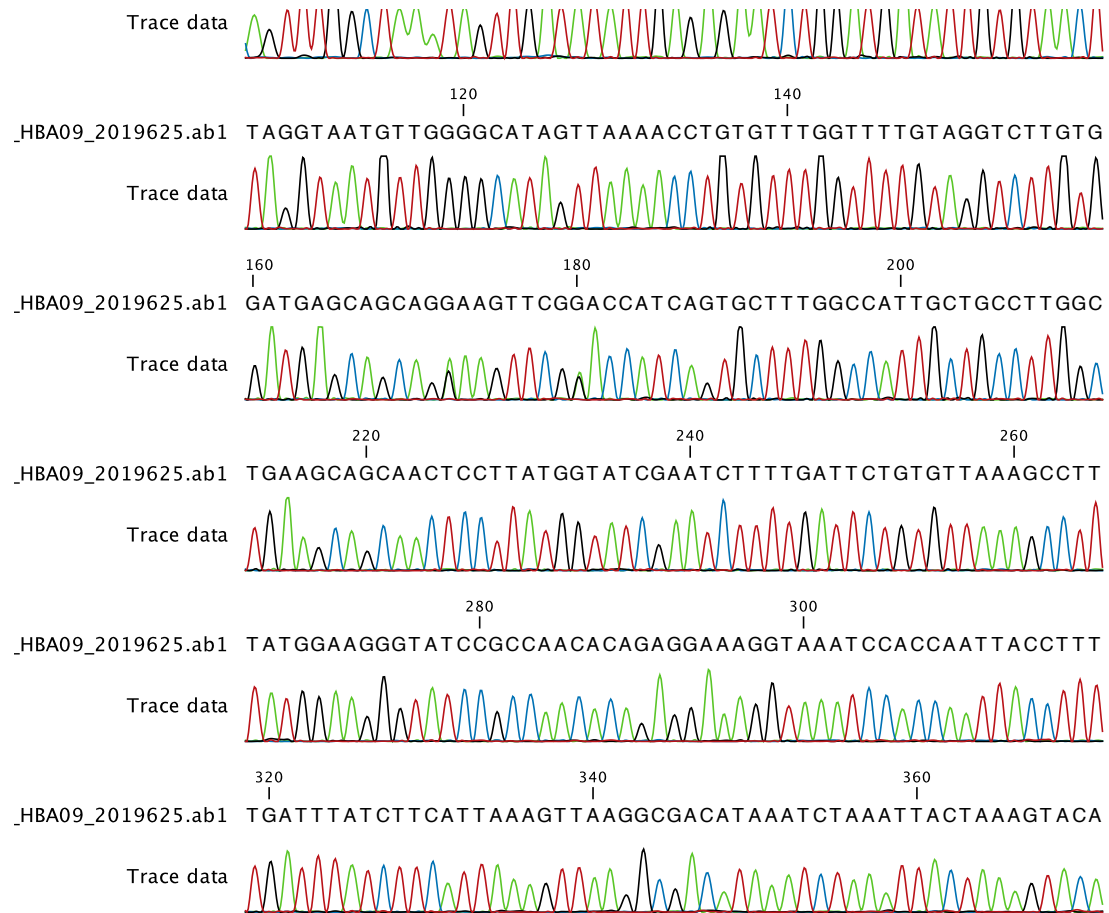

1D *SF3B1* WT

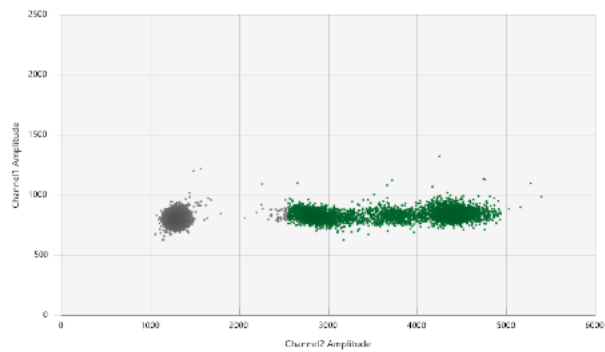

1D *SF3B1* K700E

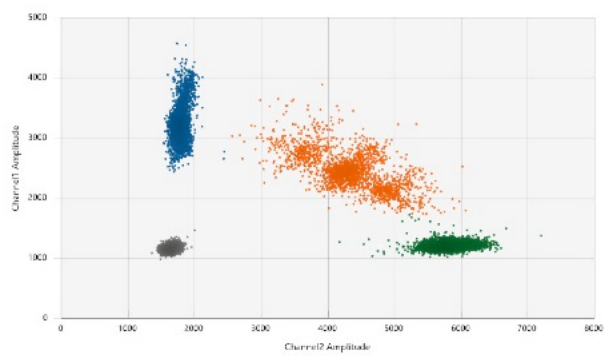

1E B-ACTIN

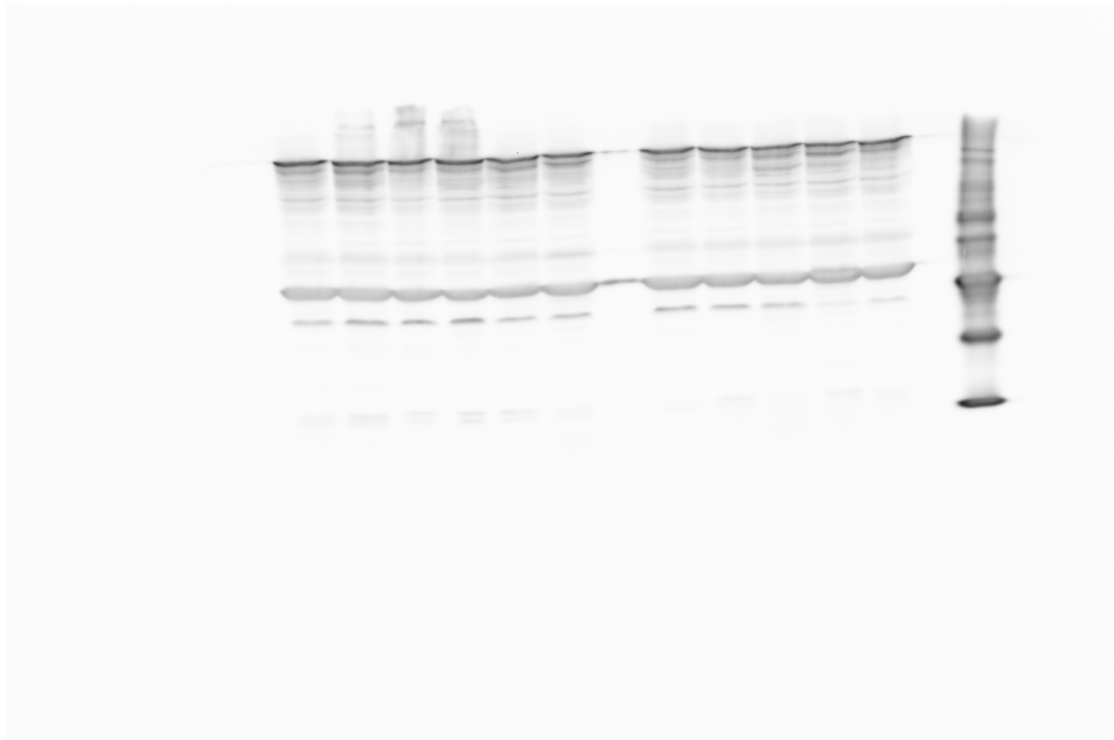

1E SF3B1

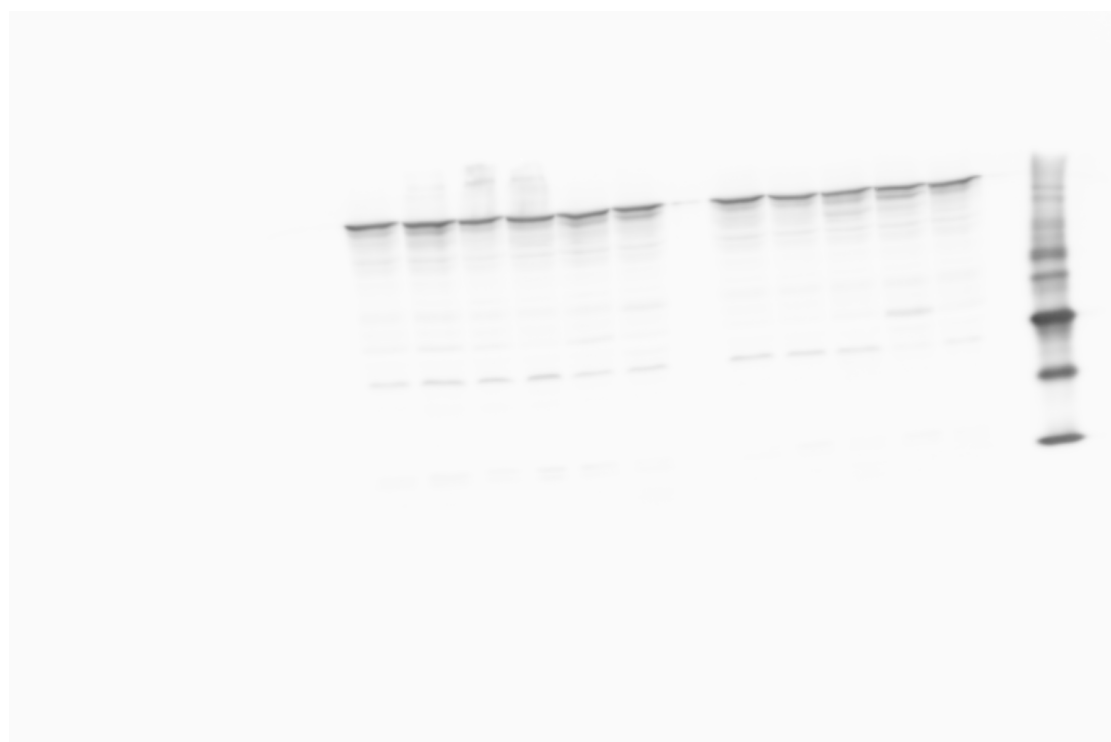

Supplement: Supplementary file 1 [file LSA-2023-01955_SdataF1.pdf]
