# Supplementary material for: SF3B1 mutation–mediated sensitization to H3B-8800 splicing inhibitor in chronic lymphocytic leukemia
Source: Life Sci Alliance. 2023 Aug 10;6(11):e202301955. doi: 10.26508/lsa.202301955 (PMC10415613; doi:10.26508/lsa.202301955)

<u>Source Data For Figure 4</u>

4E ZNF561 WT and ZNF BP1

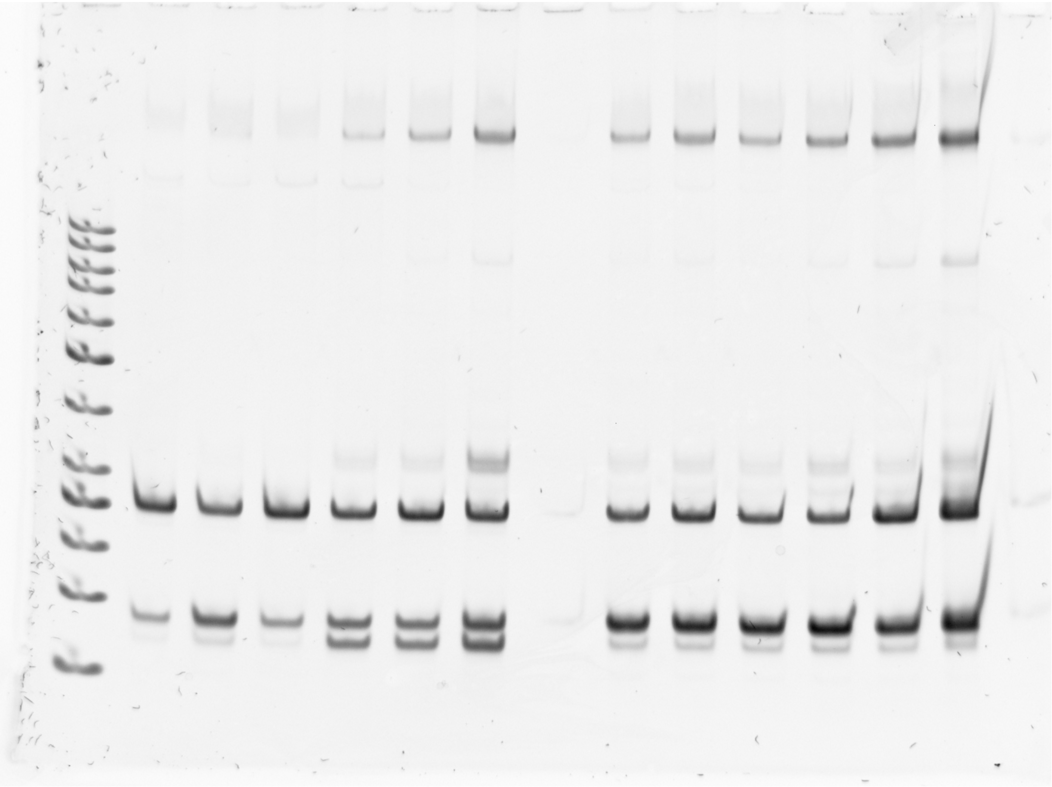

4E ZNF561 BP2

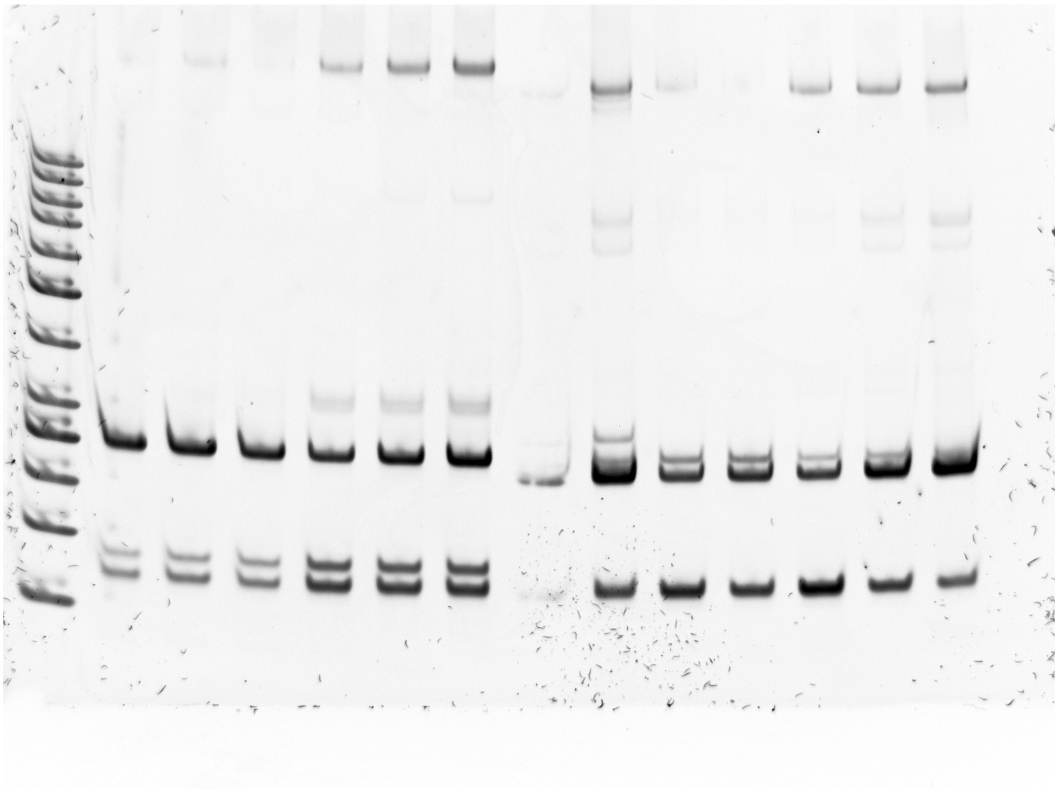

4F MAP3K7 WT

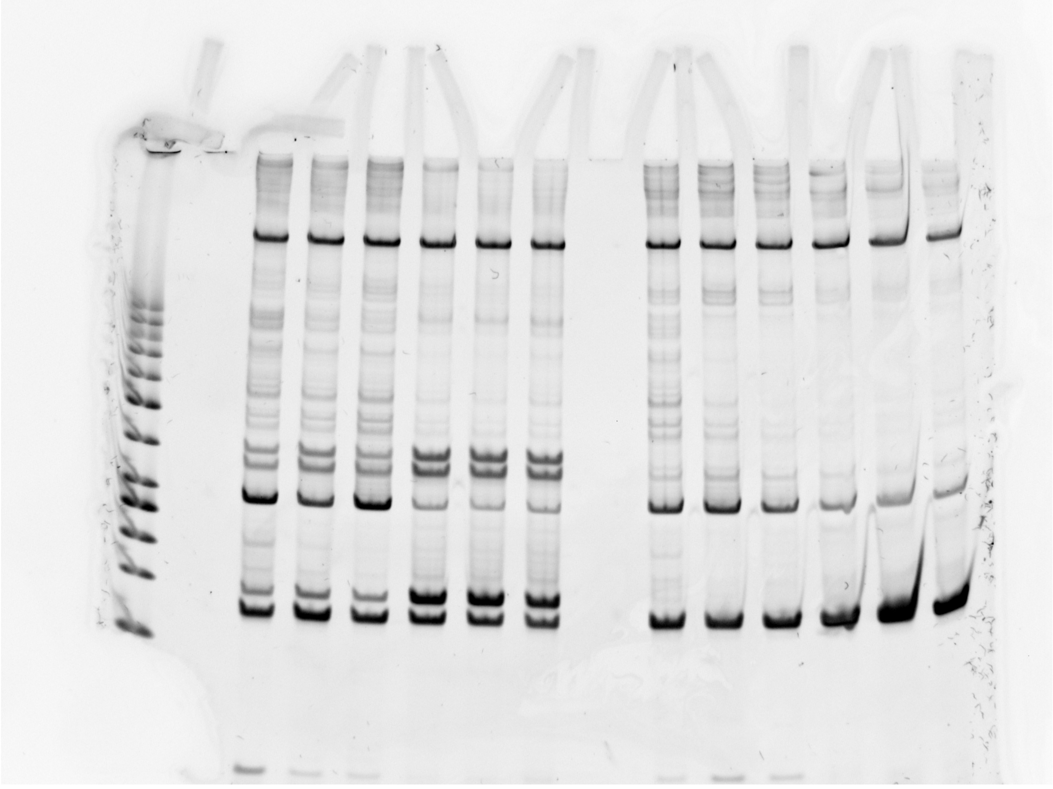

4F MAP3K7 BP1 and BP2

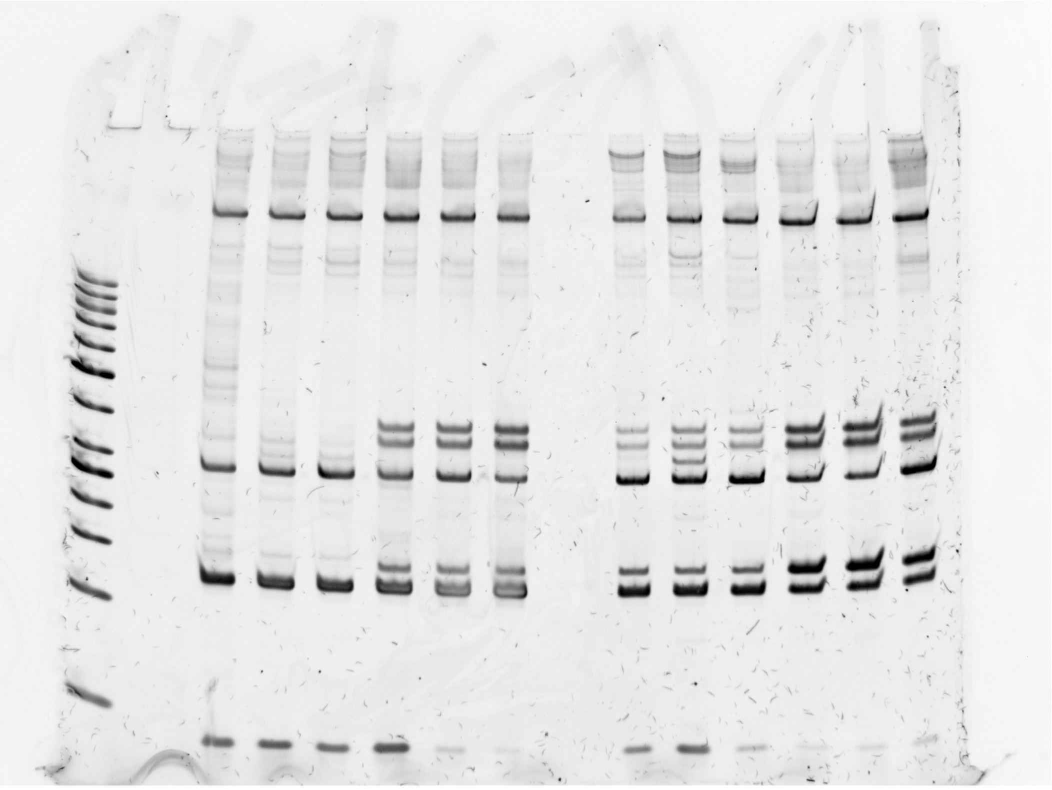

4F MAP3K7 BP 1,2,3,4

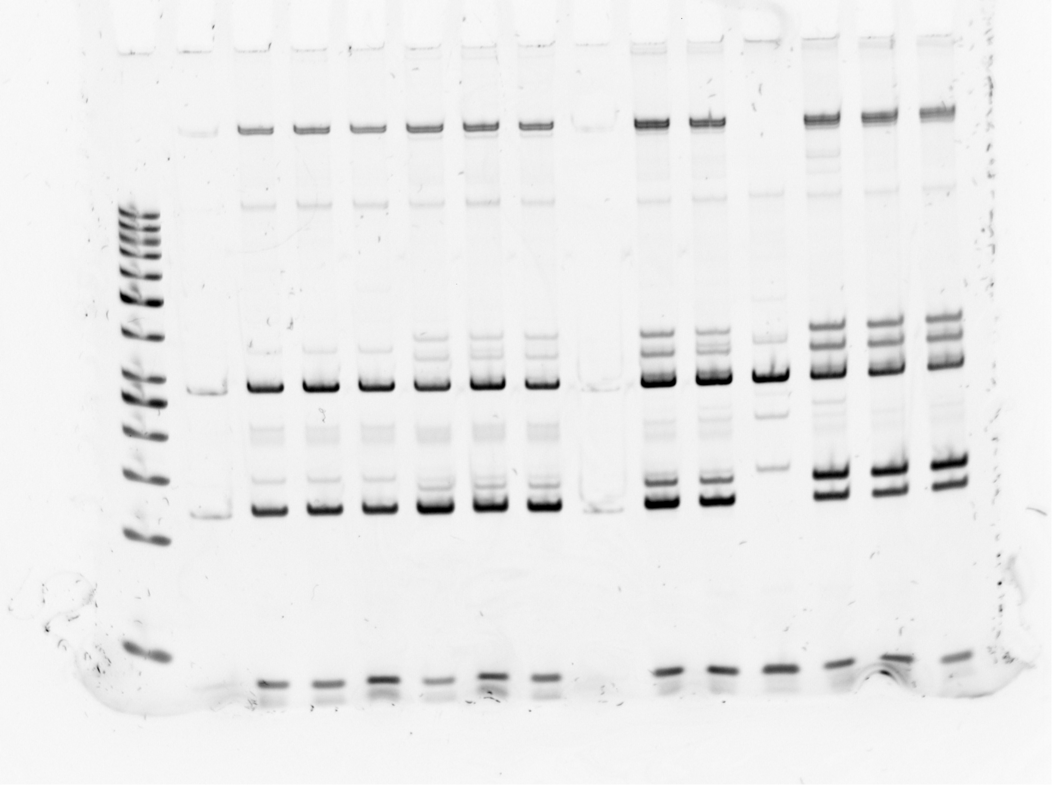

4G TARBP1 WT

Applied Biosystems
GeneMapper Software 6

19-6-23 i

Sample File	Sample Name	Panel	OS	SQ
1_236080831_HBA09.fsa	1_236080831_HBA09	None		

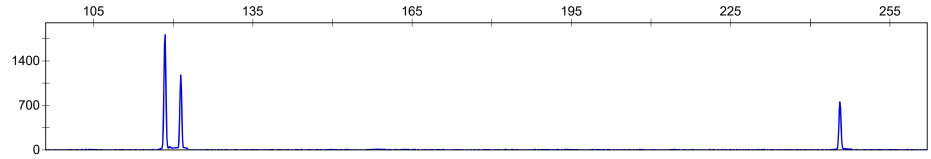

Sample File	Sample Name	Panel	OS	SQ
2_236080831_HBA09.fsa	2_236080831_HBA09	None		

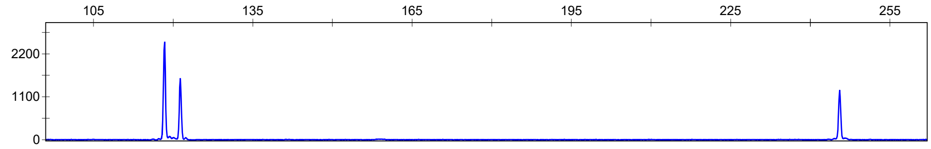

Sample File	Sample Name	Panel	OS	SQ
3_236080831_HBA09.fsa	3_236080831_HBA09	None		

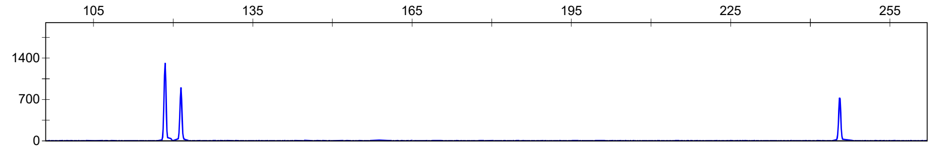

Sample File	Sample Name	Panel	OS	SQ
4_236080831_HBA09.fsa	4_236080831_HBA09	None		

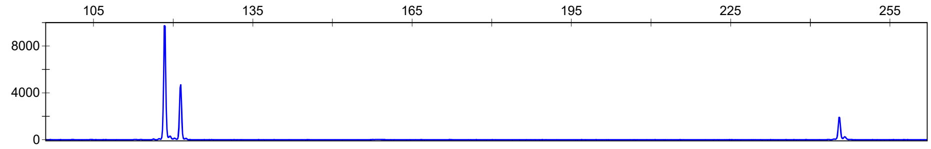

Sample File	Sample Name	Panel	OS	SQ
5_236080831_HBA09.fsa	5_236080831_HBA09	None		

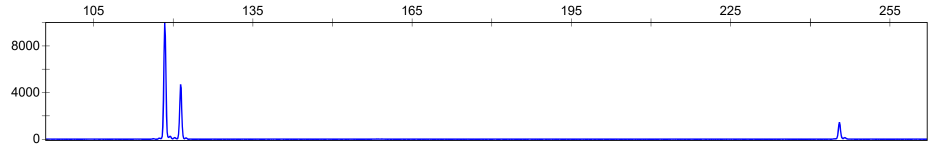

Sample File	Sample Name	Panel	OS	SQ
6_236080831_HBA09.fsa	6_236080831_HBA09	None		

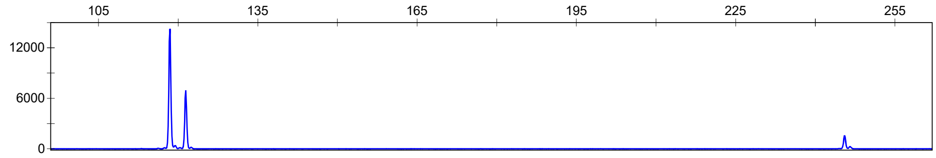

4G TARBP1 BP1

Sample File	Sample Name	Panel	OS	SQ
19_236080831_HBA09.fsa	19_236080831_HBA09	None		

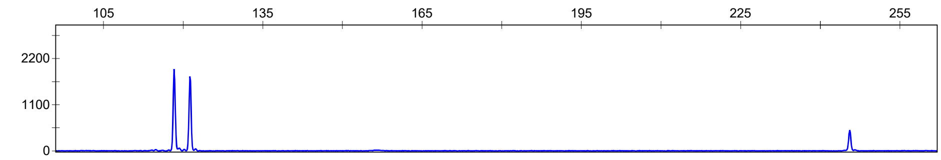

Sample File	Sample Name	Panel	OS	SQ
20_236080831_HBA09.fsa	20_236080831_HBA09	None		

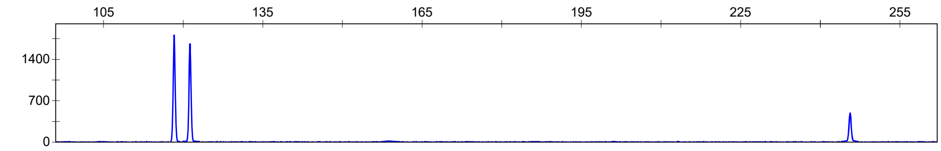

Sample File	Sample Name	Panel	OS	SQ
21_236080831_HBA09.fsa	21_236080831_HBA09	None		

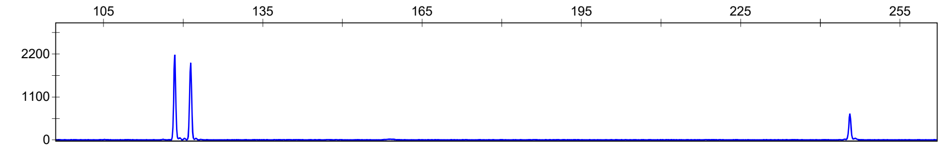

Sample File	Sample Name	Panel	OS	SQ
22_236080831_HBA09.fsa	22_236080831_HBA09	None		

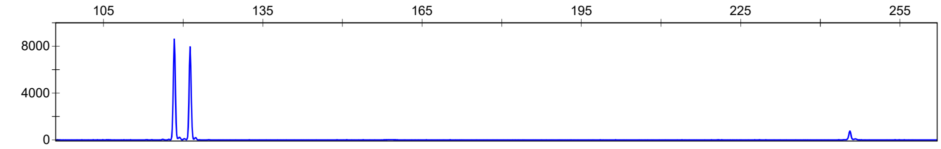

Sample File	Sample Name	Panel	OS	SQ
23_236080831_HBA09.fsa	23_236080831_HBA09	None		

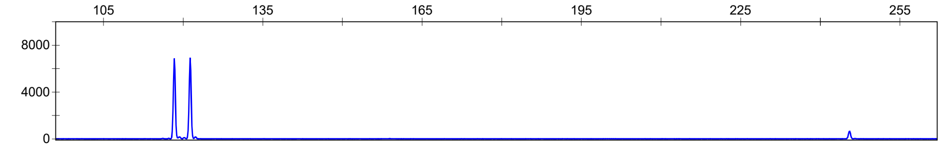

Sample File	Sample Name	Panel	OS	SQ
24_236080831_HBA09.fsa	24_236080831_HBA09	None		

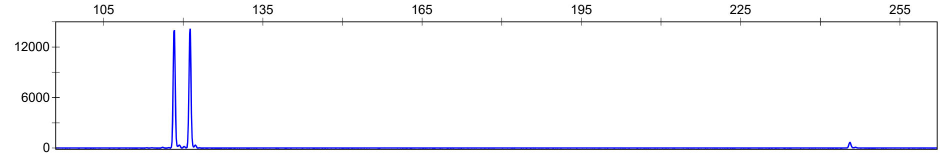

4G TARBP1 BP1

Sample File	Sample Name	Panel	OS	SQ
25_236080831_HBA09.fsa	25_236080831_HBA09	None		

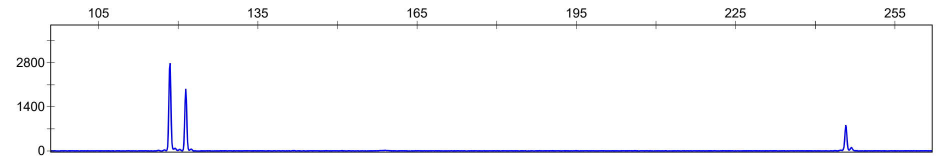

Sample File	Sample Name	Panel	OS	SQ
26_236080831_HBA09.fsa	26_236080831_HBA09	None		

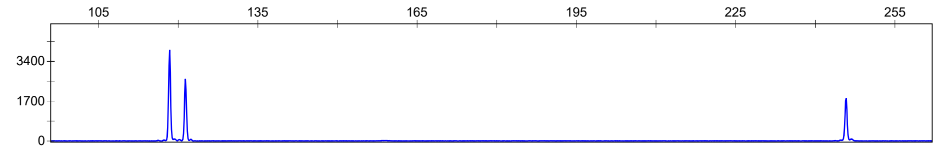

Sample File	Sample Name	Panel	OS	SQ
27_236080831_HBA09.fsa	27_236080831_HBA09	None		

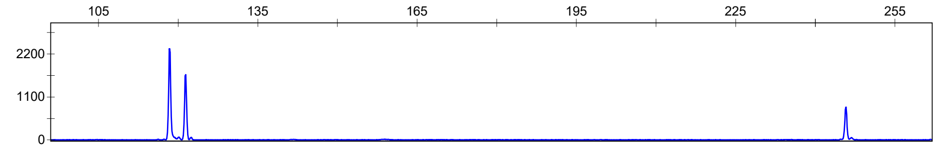

Sample File	Sample Name	Panel	OS	SQ
28_236080831_HBA09.fsa	28_236080831_HBA09	None		

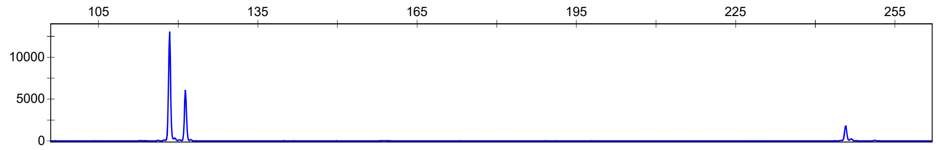

Sample File	Sample Name	Panel	OS	SQ
29_236080831_HBA09.fsa	29_236080831_HBA09	None		

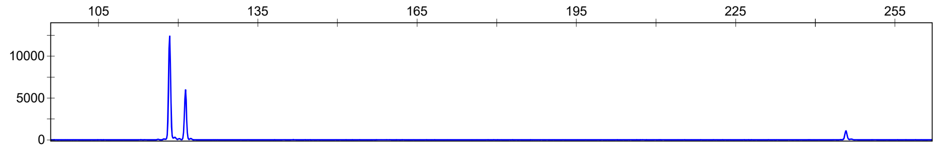

Sample File	Sample Name	Panel	OS	SQ
30_236080831_HBA09.fsa	30_236080831_HBA09	None		

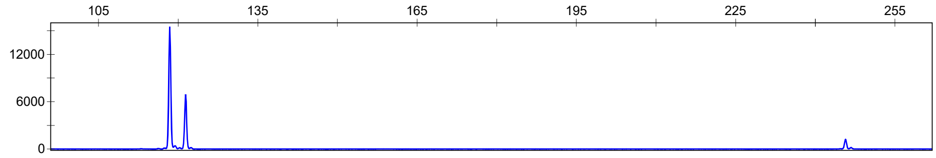

Supplement: Supplementary file 9 [file LSA-2023-01955_SdataF4.pdf]
