# Supplementary material for: SF3B1 mutation–mediated sensitization to H3B-8800 splicing inhibitor in chronic lymphocytic leukemia
Source: Life Sci Alliance. 2023 Aug 10;6(11):e202301955. doi: 10.26508/lsa.202301955 (PMC10415613; doi:10.26508/lsa.202301955)

Source Data For Figure S3

S3 A INPPL1

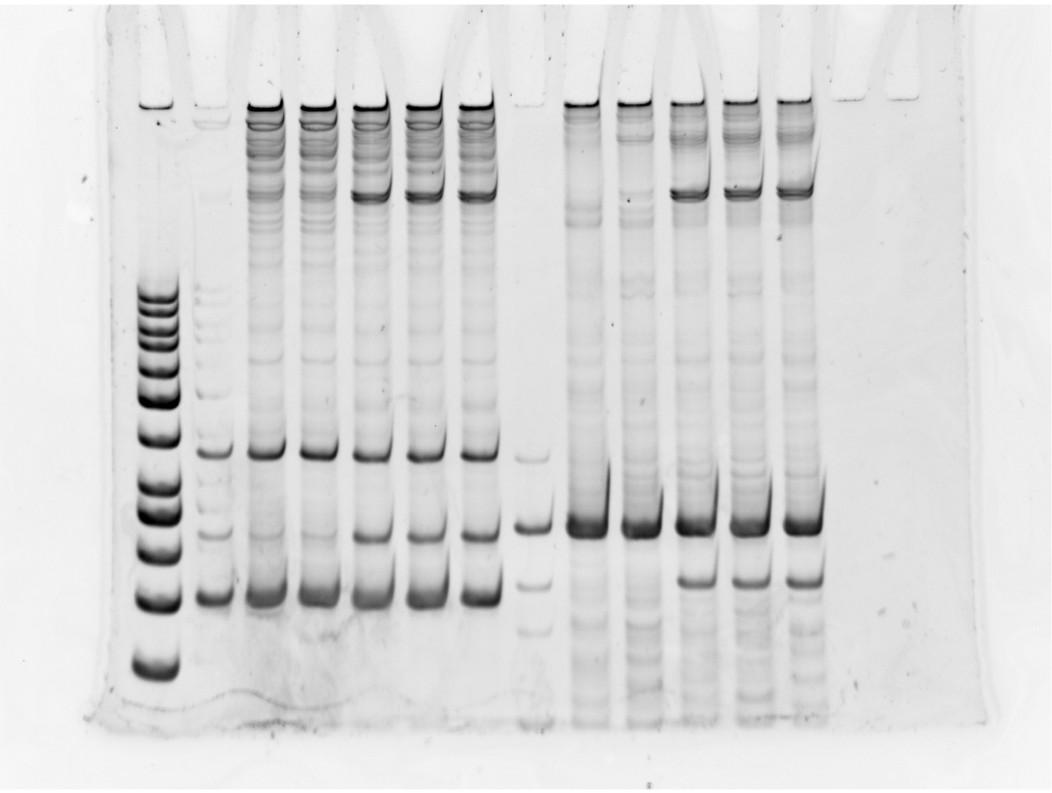

S3 A DLG1

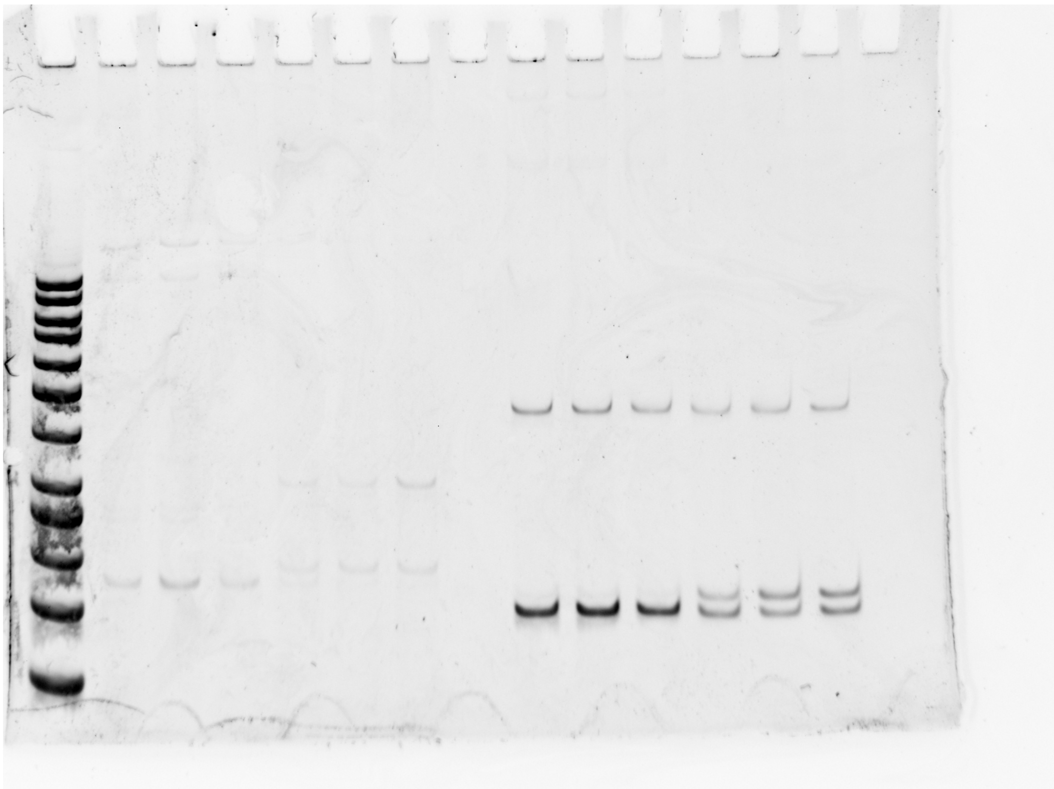

S3 A DYNLL1

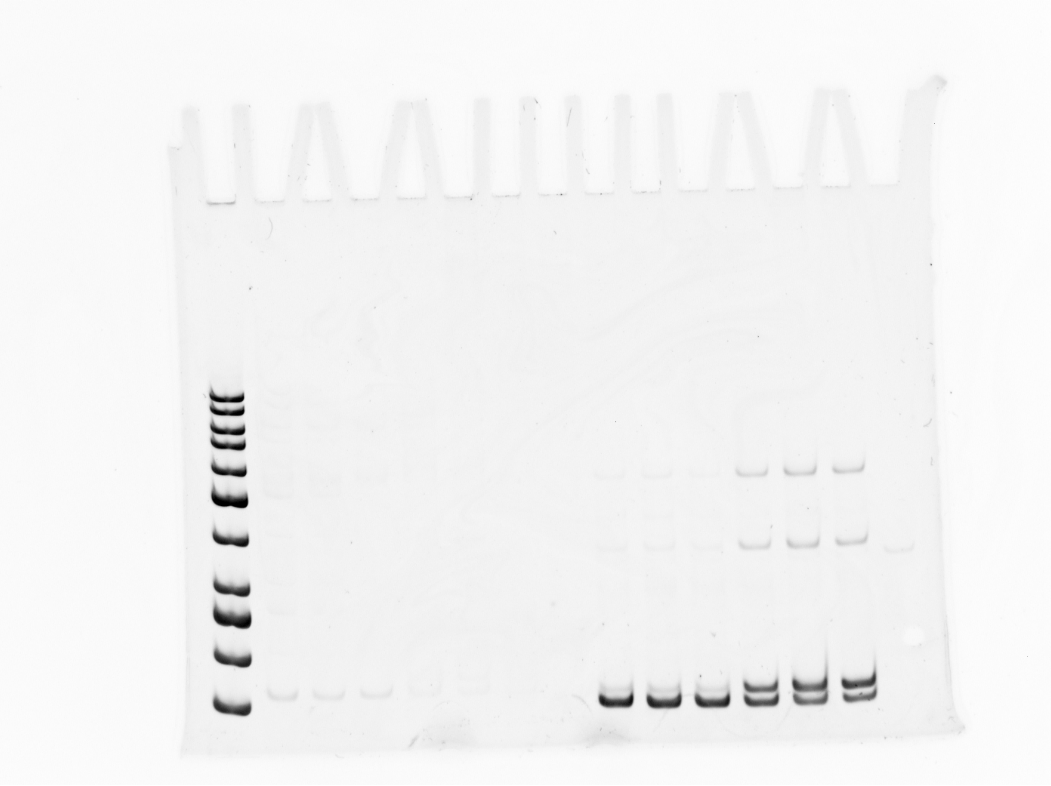

S3 A HLTF

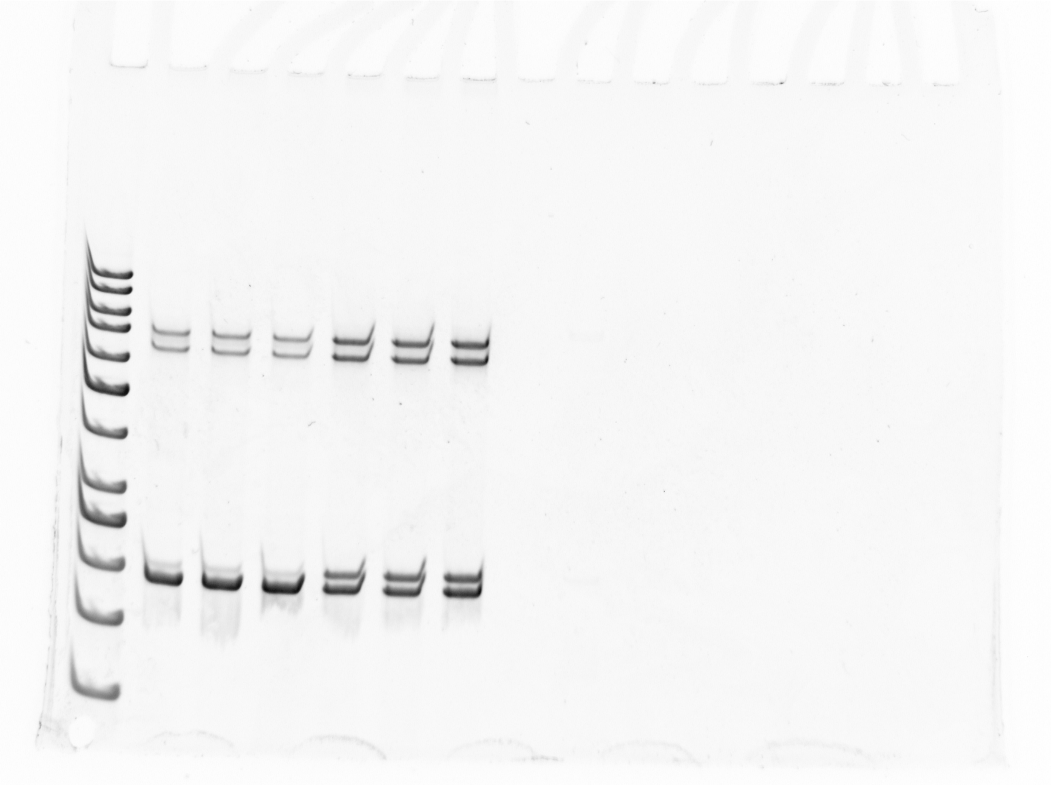

S3 A MAP3K7

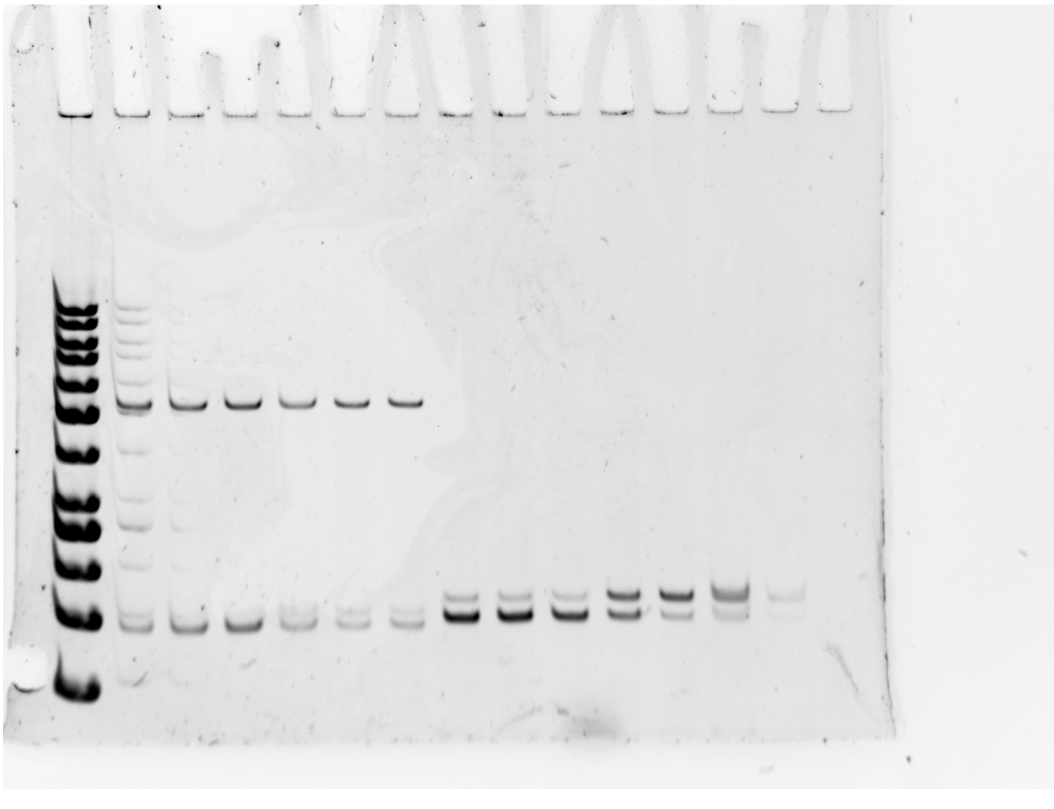

S3 A TGFBR1

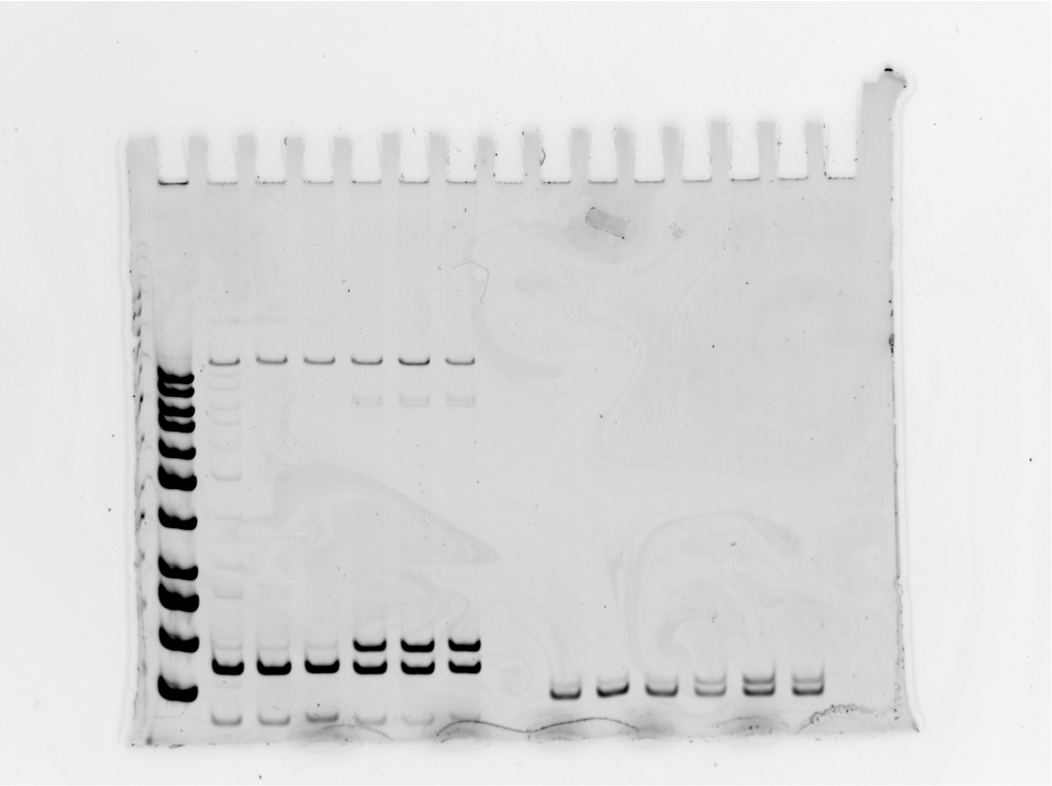

S3 A UBA7

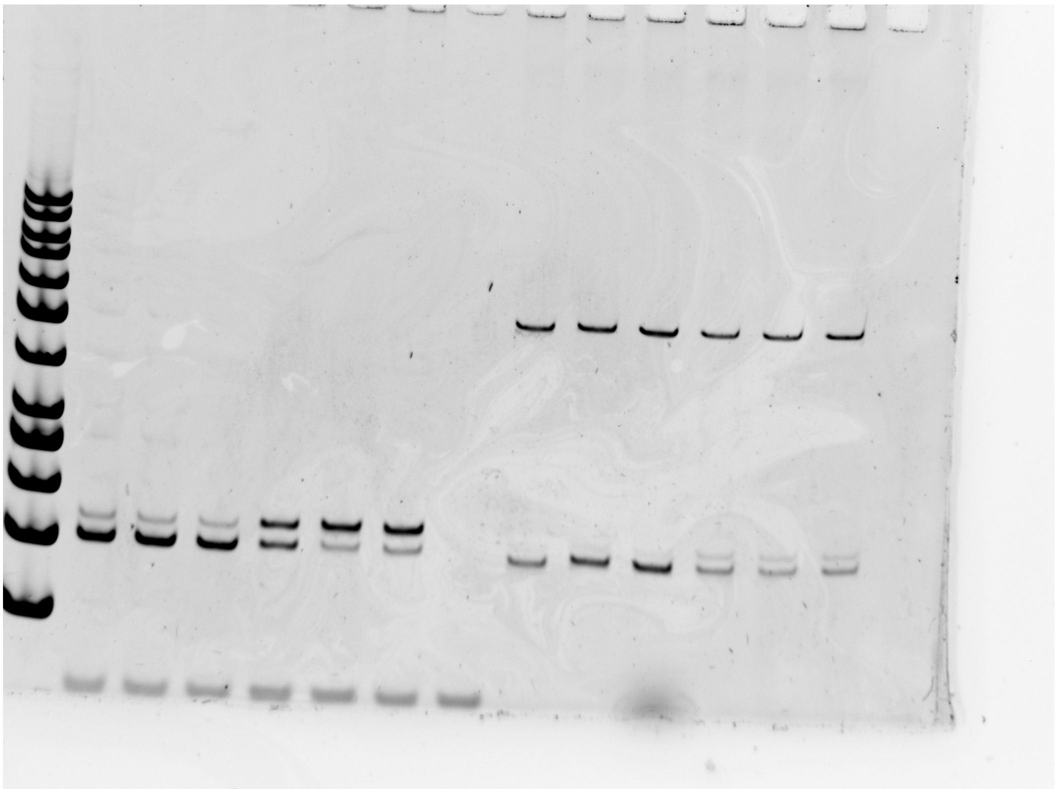

S3 A ZDHHC16

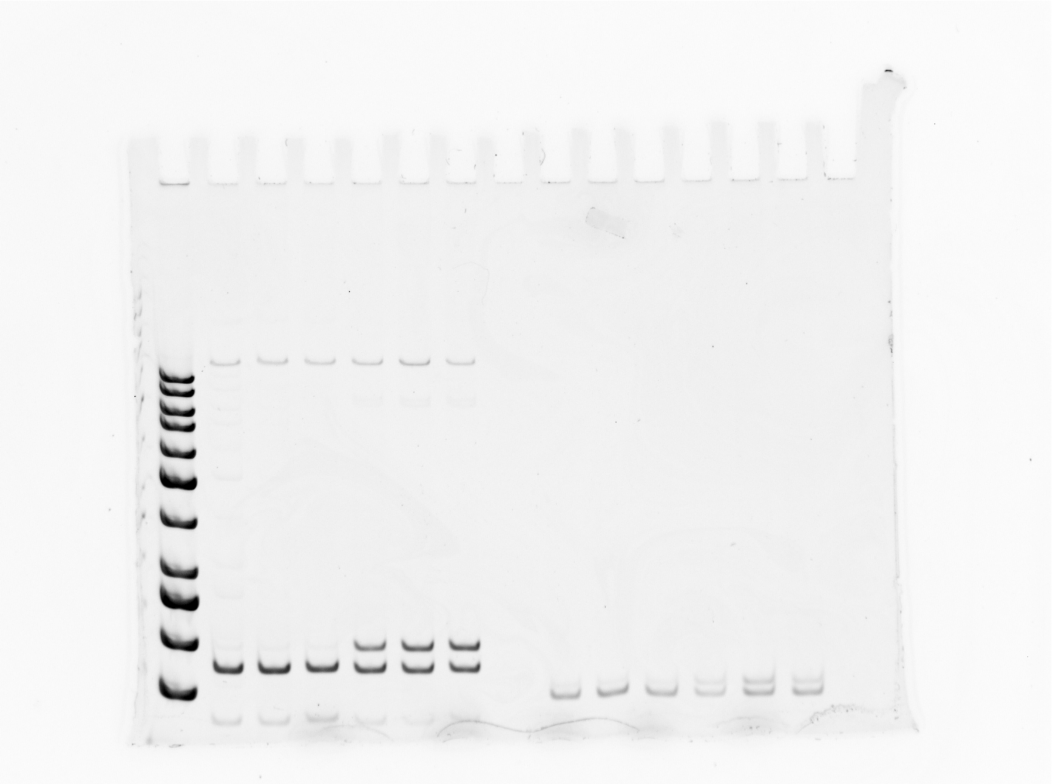

S3 A ANKHD1

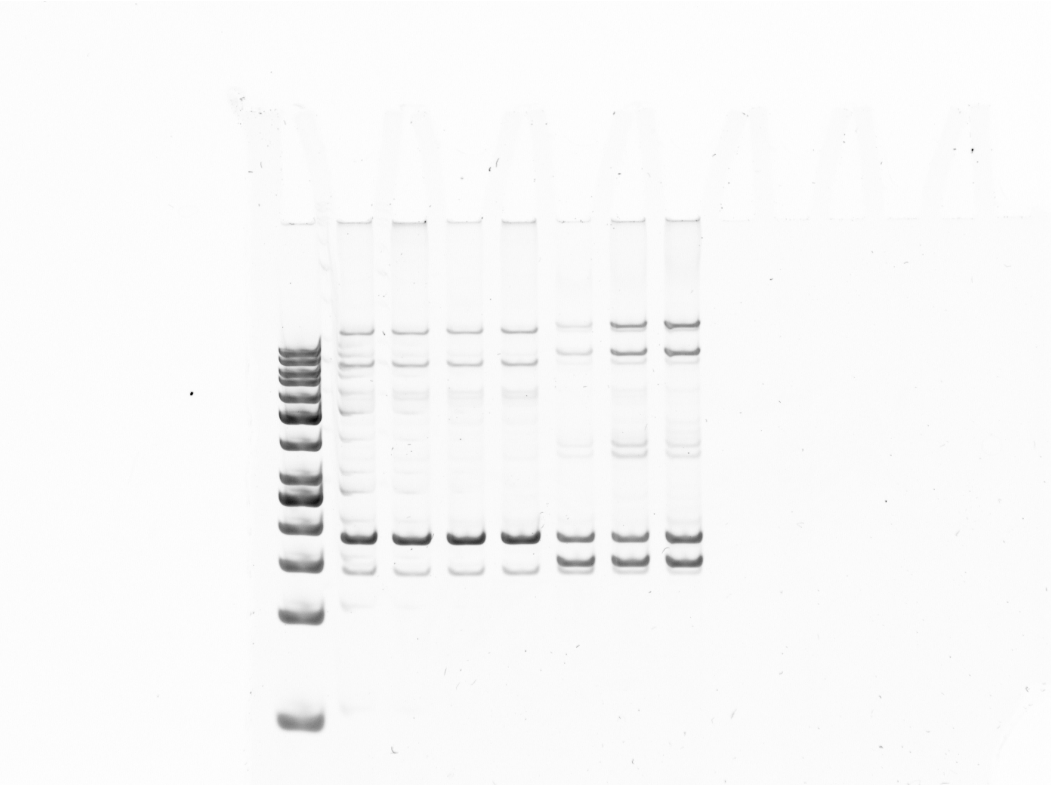

S3 A NFKB1 and ZNF561

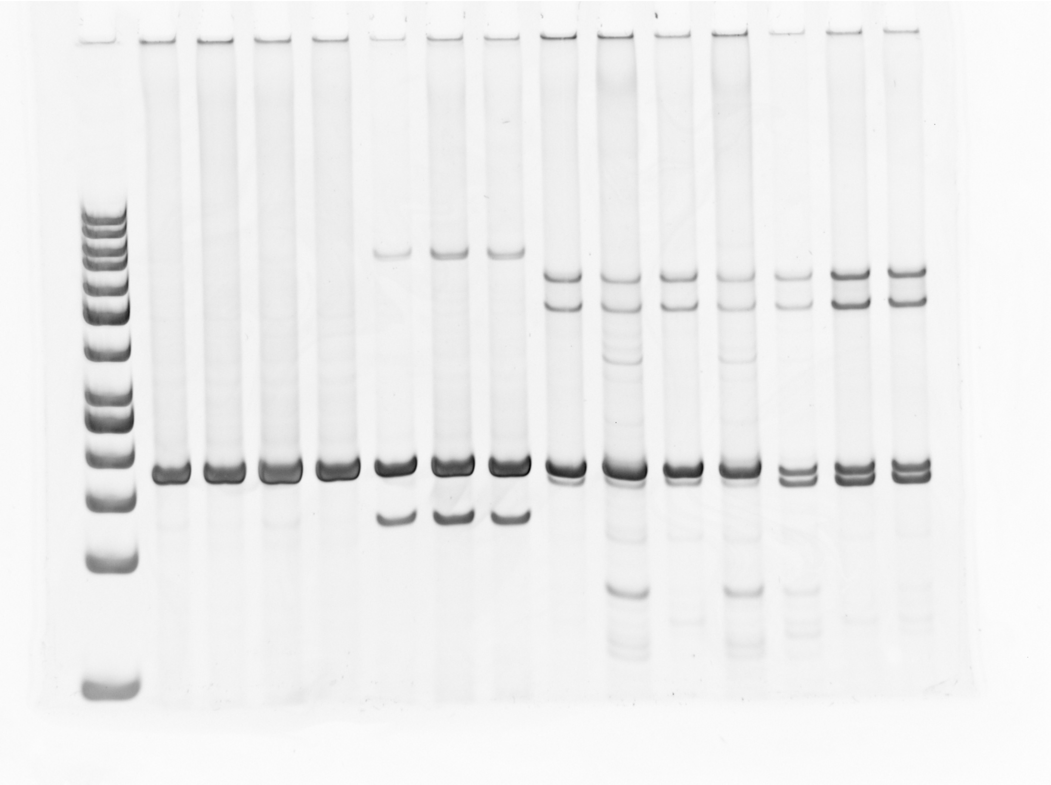

S3 A FBXO41

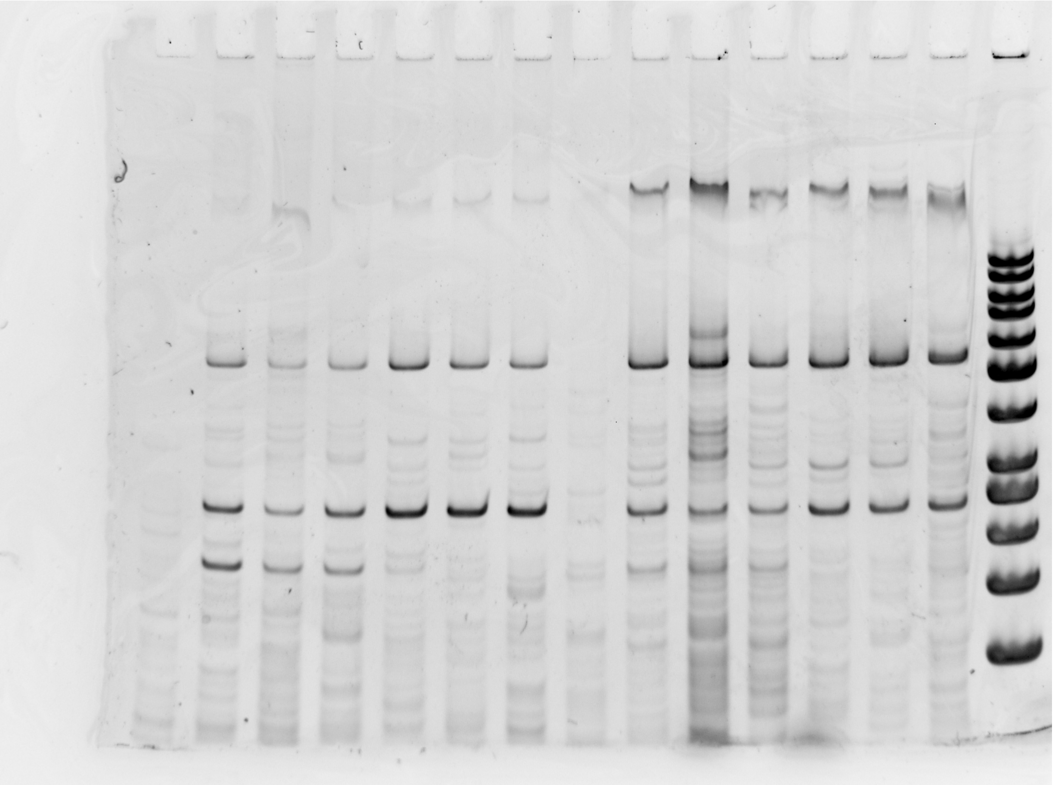

Supplement: Supplementary file 12 [file LSA-2023-01955_SdataFS3.pdf]
