# Supplementary material for: SF3B1 mutation–mediated sensitization to H3B-8800 splicing inhibitor in chronic lymphocytic leukemia
Source: Life Sci Alliance. 2023 Aug 10;6(11):e202301955. doi: 10.26508/lsa.202301955 (PMC10415613; doi:10.26508/lsa.202301955)

Source Data For Figure S5

S5 MAP3K7 WT and MAP3K7 BP1,5

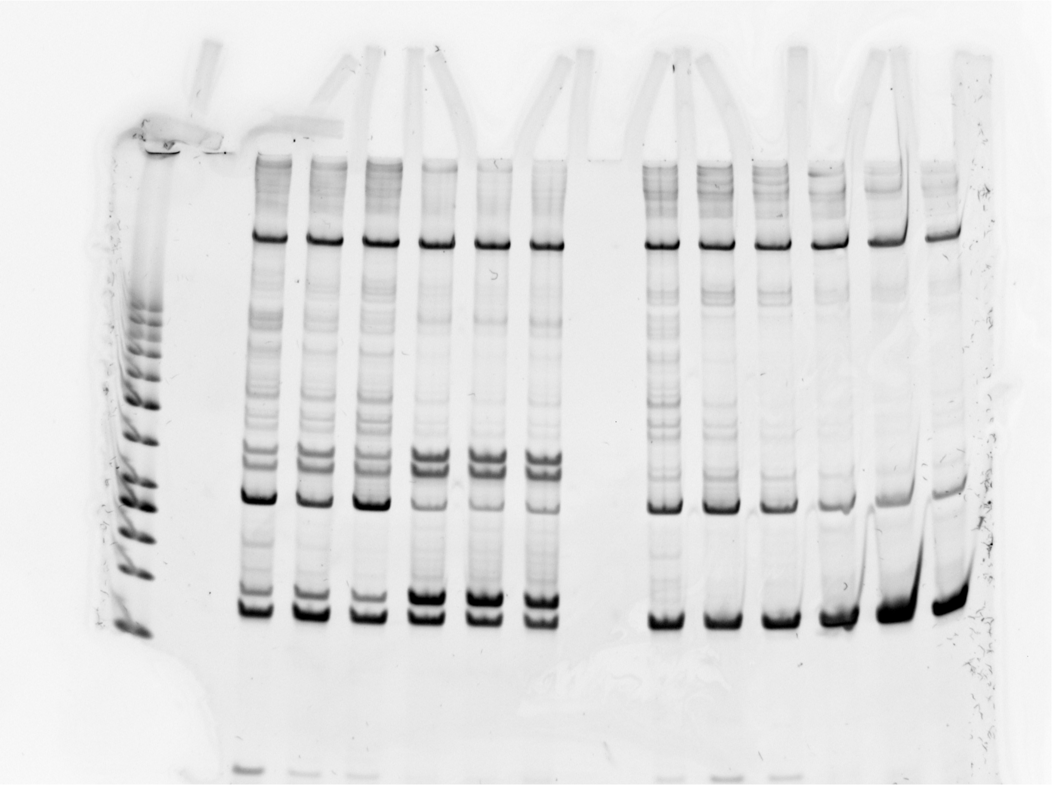

S5 MAP3K7 BP1 and BP2

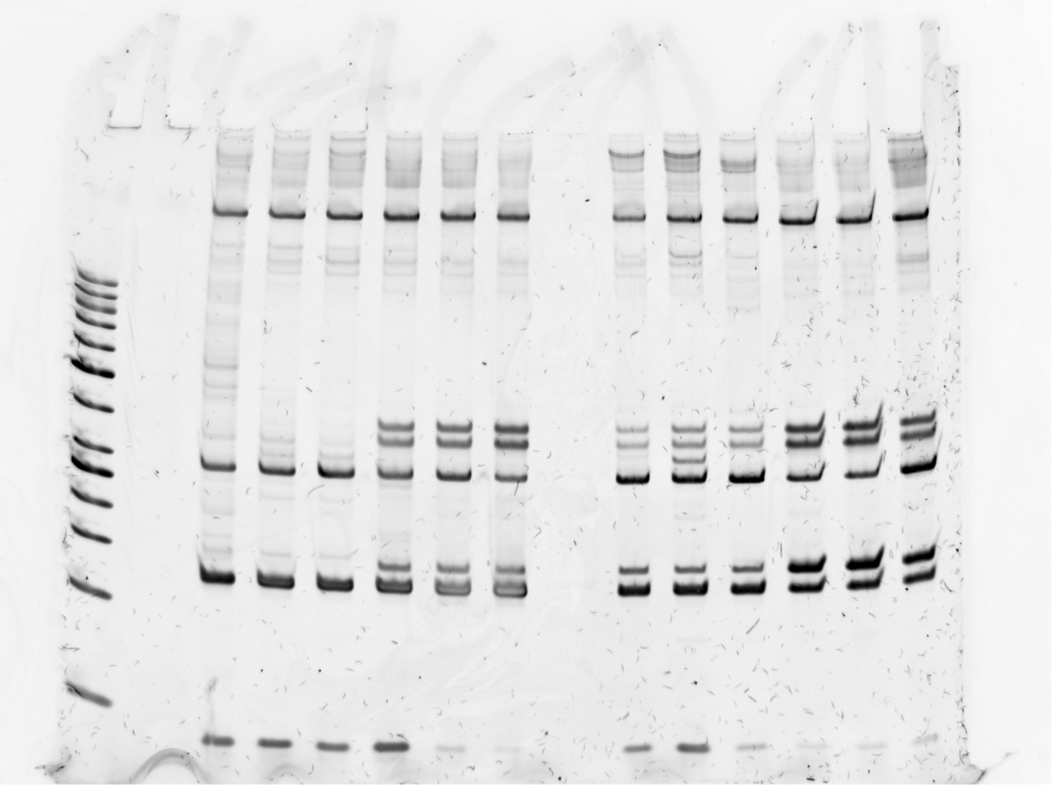

S5 MAP3K7 BP3

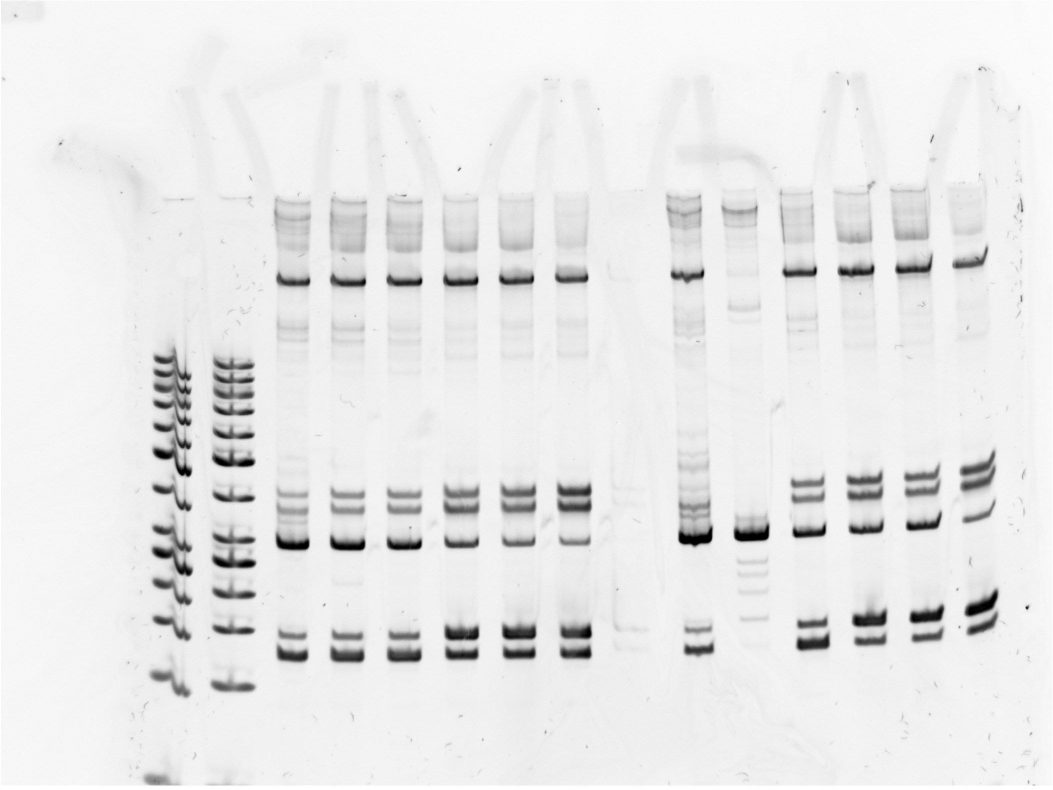

S5 MAP3K7 BP4

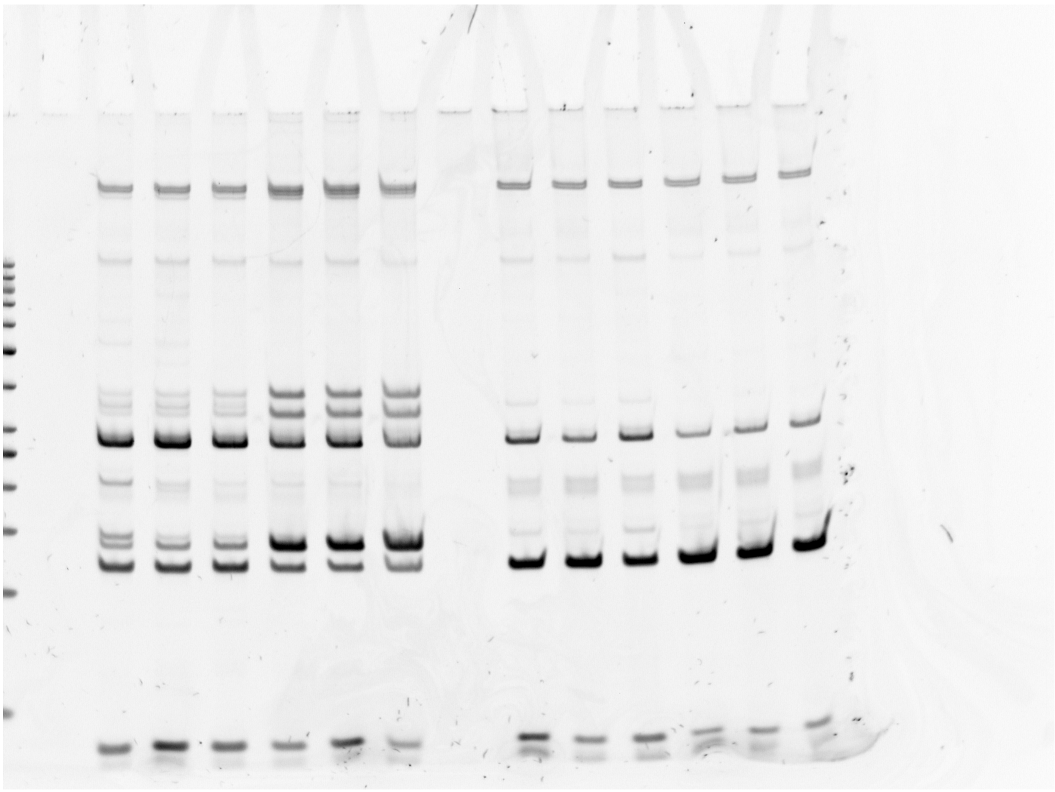

S5 MAP3K7 BP5

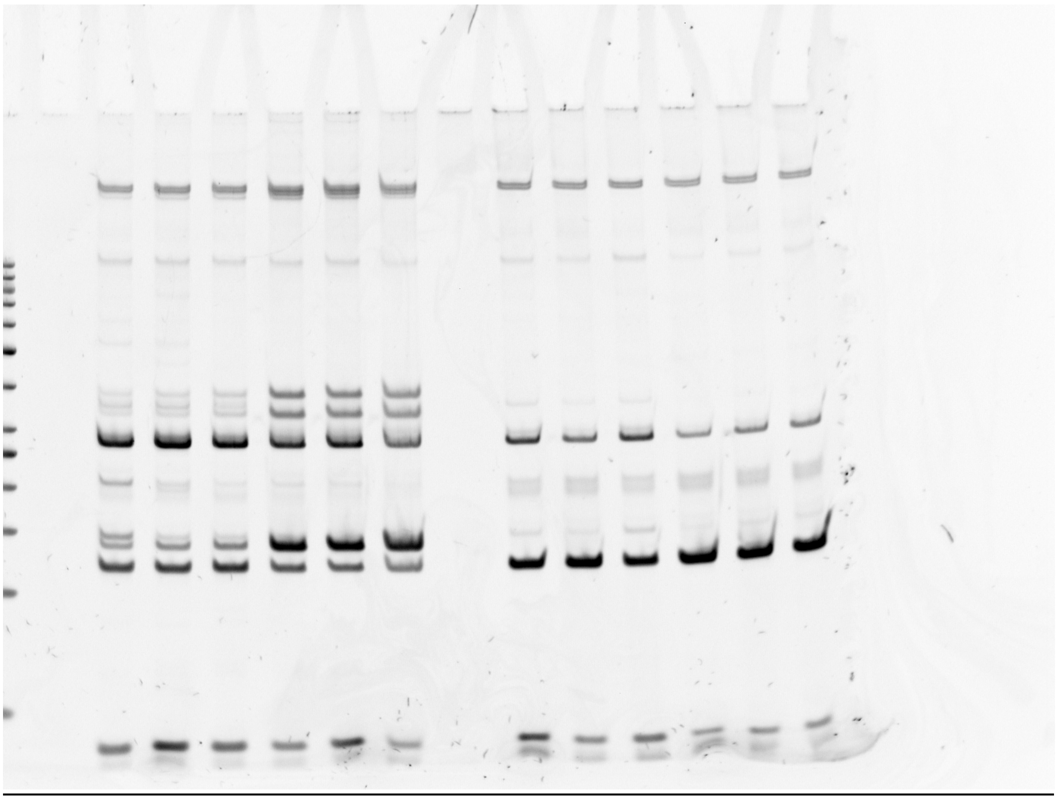

S5 MAP3K7 BP1,2,3,4

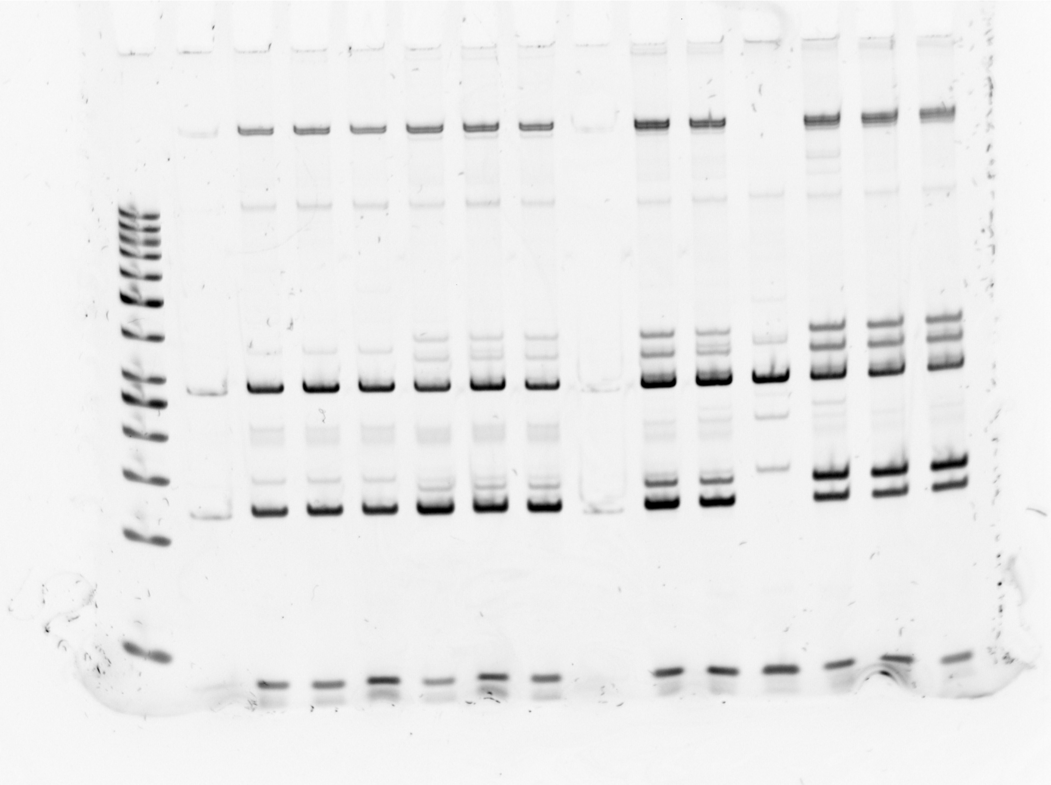

S5 MAP3K7 BP1,2,3,4,5

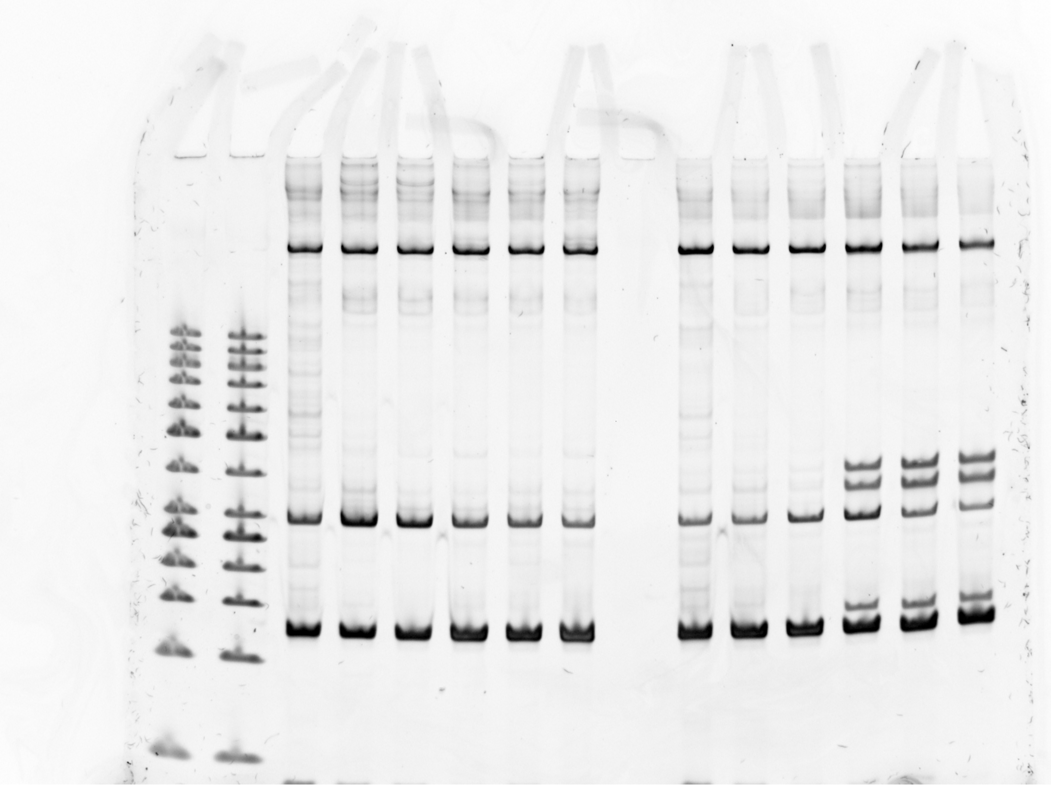

Supplement: Supplementary file 15 [file LSA-2023-01955_SdataFS5.pdf]
